# Supplementary material for: Tumor Treating Fields Concomitant with Sorafenib in Advanced Hepatocellular Cancer: Results of the HEPANOVA Phase II Study
Source: Cancers (Basel). 2022 Mar 18;14(6):1568. doi: 10.3390/cancers14061568 (PMC8946145; doi:10.3390/cancers14061568)

Supplemental Material

Eligibility Criteria

Inclusion Criteria:

- Hepatocellular cancer (HCC) diagnosed by biopsy, or by imaging criteria (computed tomography/magnetic resonance imaging) and alpha-fetoprotein.
- ≥ 18 years of age.
- Barcelona Clinic Liver Cancer Staging Stage 0–C.
- Child-Turcotte-Pugh score between 5 and 8 points.
- Measurable disease per Response Evaluation Criteria in Solid Tumors criteria for HCC.
- ≥ 4 weeks since major surgery.
- Eastern Cooperative Oncology Group performance status of 0–2.
- Life expectancy of ≥ 12 weeks.
- All patients must sign written informed consent.
- Able to operate the NovoTTF-100L(P) System independently or with the help of a caregiver.

Exclusion Criteria:

- Patient candidate for surgical resection or local treatment (e.g., transcatheter arterial chemoembolization, selective internal radiation therapy, radiofrequency-

induced thermo-ablation, microwave, surgery).

- High levels of serum hepatitis B viral DNA without anti-viral therapy.
- Prior malignancy requiring anti-tumor treatment (apart from *in-situ* cervical cancer, in situ breast cancer, non-melanomatous skin cancers, or any malignancy for which treatment was received and there is no evidence of disease for ≥ 5 years) or concurrent malignancy.
- Significant comorbidities within 4 weeks prior to enrollment, including the following:
  - Neurologic or psychiatric disorders such as dementia and uncontrolled seizures.
  - Active, uncontrolled infections.
  - Active, disseminated coagulation disorder.
  - Significant renal impairment.
  - Clinically significant cardiovascular disease (including myocardial infarction, unstable angina, symptomatic congestive heart failure, serious uncontrolled cardiac arrhythmia).
  - Chronic diarrhea.
  - History of any psychological or psychiatric condition that might impair the patient's ability to understand or comply with the requirements of the study or to provide consent.

- Clinically significant (as determined by the investigator) hematologic or renal dysfunction, defined as: hemoglobin < 10 g/dL, neutrophil count < 1.5 x 10^9/L, platelet count < 100 x 10^9/L, or serum creatinine > 1.5 x upper limit of normal.
- Other comorbidities deemed by the investigator as such that may impact the compliance of the patient with the study protocol and follow-up.

- Implanted pacemaker, defibrillator, or other electrical medical devices in the torso.
- Known allergies to medical adhesives or hydrogel.
- Pregnant or breast feeding (all patients of childbearing potential must use highly effective contraception method during the entire period of the study based on the recommendation of the investigator or a gynecologist).
- Admitted to an institution by administrative or court order.

The name of all Ethics Committees who reviewed and approved the protocol and the relevant approval number and date

Country	Hospital & Central Ethics Committee
France	CHU Nantes, Hôtel-Dieu<br>Service hépato-gastro-entérologie<br>1, place Alexis Ricordeau<br>44093 NANTES Cedex 01<br>Comité de Protection des Personnes OUEST IV – NANTES<br>Immeuble Cap Ouest<br>Maison de la Recherche en Santé<br>53, Chaussée de la Madeleine 44000 NANTES<br>Central Ethics Commitee approval: 28.06.2018<br>Ministry of Health approval: 07.06.2018
Germany	Medical Center – University of Freiburg, Center for Diagnostic and Therapeutic Radiology<br>Department of Radiation Oncology, Robert-Koch-Straße 3 79106 Freiburg<br>Ethik-Kommission der Albert-Ludwigs-Universität Freiburg<br>Engelberger Straße 21<br>79106 Freiburg<br>Ethics Committee approval: 23.05.2019<br>Ministry of Health approval: 30.11.2018
Germany	Ulm University Hospital<br>Albert-Einstein-Allee 23<br>D-89081 Ulm/Germany<br>Coordinating Ethics Committee:<br>Ethik-Kommission der Albert-Ludwigs-Universität Freiburg<br>Engelberger Straße 21<br>79106 Freiburg

Country	Hospital & Central Ethics Committee
	Ethics Committee approval: 22.10.2019<br><br>Ministry of Health approval: 23.08.2019
Spain	Vall d´Hebrón University Hospital<br><br>pg Vall d´Hebrón 119-129,<br><br>08035 Barcelona<br><br>CEIC Hospital Universitari Vall d'Hebron<br><br>pg Vall d´Hebrón 119-129,<br><br>08035 Barcelona<br><br>Central Ethics Committee approval: 06.04.2018<br><br>Ministry of Health approval: 14.05.2018
Spain	HM Universitario Sanchinarro,<br><br>CIOCC, C/Oña no. 10, Madrid 28050, Spain<br><br>CEIC Hospital Universitari Vall d'Hebron<br><br>pg Vall d´Hebrón 119-129,<br><br>08035 Barcelona<br><br>Central Ethics Committee approval: 04.05.2018<br><br>Ministry of Health approval: 14.05.2018
Poland	Clinical Research Center Piotr Napora Doctors Partner Company, ul. Dlugosza 4 51-162 Wroclaw, Poland<br><br>Coordinating EC:<br><br>Komisja Bioetyczna przy Uniwersytecie Medycznym<br><br>im. Karola Marcinkowskiego w Poznaniu<br><br>Centrum Stomatologii<br><br>ul. Bukowska 70, pok A204<br><br>60-812 Poznań<br><br>Central Ethics Committee approval: 12.09.2019<br><br>Ministry of Health approval: 14.01.2020

Table S1. Investigational study sites.

Country	Study site
France	CHU Hôtel-Dieu, Nantes
Germany	Medical Center – University of Freiburg, Freiburg
Germany	Ulm University Hospital, Ulm
Poland	Clinical Research Center Piotr Napora Doctors Partner Company
Spain	Vall d'Hebron Institute of Oncology (VHIO), Barcelona
Spain	HM Sanchinarro University Hospital, Madrid

Table S2. Results from the literature search used to calculate the historical control ORR for sorafenib.

Reference	*N*	Regimen	ORR (sorafenib)
Llovet JM et al	299	Sorafenib 400 mg twice daily	2.0%
Abou-Alfa GK et al	137	Sorafenib 400 mg twice daily	2.2%
Zhu AX et al	358	Sorafenib 400 mg twice daily	3.9%
Cainap C et al	521	Sorafenib 400 mg twice daily	6.9%

ORR = overall response rate.

Table S3. Response in all evaluable patients treated with TTFields (150 kHz) concomitant with sorafenib, according to CTP class.

**Outcome**	**CTP class A (*n* = 11)**	**CTP class B (*n* = 10)**	**Historical control***
Overall response rate, %	9.1	10	4.5
Level of response rate, %			
Complete	0	0	–
Partial	9.1	10	–
Stable disease	63.6	70	–
Disease control rate, %	73	80	–
In-field control rate at 1 year, %	9.1	10	–

* See Table S2. CTP = Child-Turcotte-Pugh; TTFields = Tumor Treating Fields.

Table S4. Time to event outcomes with TTFields (150 kHz) concomitant with sorafenib, according to CTP class.

**Outcome**	**CTP class A (*n* = 11)**	**CTP class B (*n* = 10)**
OS rate at 1 year, % (95% CI)	29 (17–68)	27 (7–51)
PFS rate at 12 months, % (95% CI)	29 (5–61)	20 (3–47)
Distant metastases-free survival rate at 1 year, % (95% CI)	38 (5–72)	20 (3–47)
Median time to progression, months (95% CI)	8.9 (3.0–not reached)	6 (0.9–not reached)

CI = confidence interval; CTP = Child-Turcotte-Pugh; OS = overall survival; PFS = progression-free survival; TTFields = Tumor Treating Fields.

Figure S1. Skin Grade 2 skin adverse events experienced by male patient.

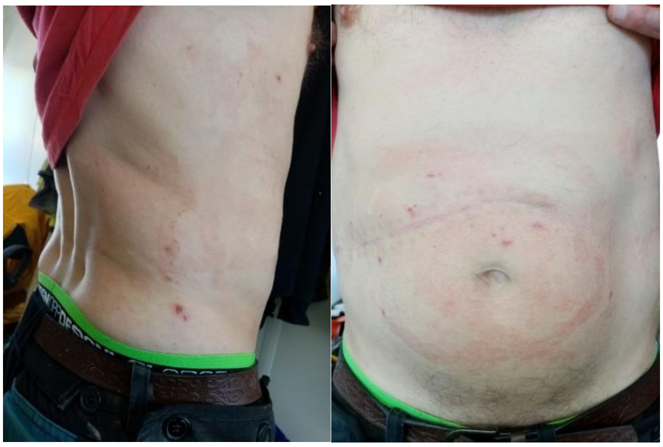

Supplement: Supplementary file 1 [file cancers-14-01568-s001.zip › cancers-1602409-supplementary.pdf]
